# Blunted Reducing Power Generation in Erythrocytes Contributes to Oxidative Stress in Prepubertal Obese Children with Insulin Resistance

**DOI:** 10.3390/antiox10020244

**Published:** 2021-02-05

**Authors:** Álvaro González-Domínguez, Francisco Visiedo, Jesus Domínguez-Riscart, Beatriz Ruiz-Mateos, Ana Saez-Benito, Alfonso M. Lechuga-Sancho, Rosa María Mateos

**Affiliations:** 1 Inflammation, Nutrition, Metabolism and Oxidative Stress Study Group (INMOX), Biomedical Research and Innovation Institute of Cádiz (INiBICA), Research Unit, Puerta del Mar University Hospital, 11009 Cádiz, Spain; alvaro.gonzalez@inibica.com (Á.G.-D.); visiedofm@hotmail.com (F.V.); jesus.dominguezriscart@gmail.com (J.D.-R.); beatriz.rmb95@gmail.com (B.R.-M.); 2 Pediatric Endocrinology and Diabetes, Department of Pediatrics, Puerta del Mar University Hospital, 11009 Cádiz, Spain; 3 Clinical Analysis Department, Puerta del Mar University Hospital, 11009 Cádiz, Spain; anasaezbenito@gmail.com; 4 Area of Pediatrics, Department of Child and Mother Health and Radiology, Medical School, University of Cádiz, 11003 Cádiz, Spain; 5 Area of Biochemistry and Molecular Biology, Department of Biomedicine, Biotechnology and Public Health, University of Cádiz, 11519 Cádiz, Spain

**Keywords:** antioxidants, childhood obesity, insulin resistance, oxidative stress, pentose phosphate pathway, reducing power

## Abstract

Childhood obesity, and specifically its metabolic complications, are related to deficient antioxidant capacity and oxidative stress. Erythrocytes are constantly exposed to multiple sources of oxidative stress; hence, they are equipped with powerful antioxidant mechanisms requiring permanent reducing power generation and turnover. Glucose-6-phosphate dehydrogenase (G6PDH) and 6-phosphogluconate dehydrogenase (6PGDH) are two key enzymes on the pentose phosphate pathway. Both enzymes supply reducing power by generating NADPH, which is essential for maintaining the redox balance within the cell and the activity of other antioxidant enzymes. We hypothesized that obese children with insulin resistance would exhibit blunted G6PDH and 6PGDH activities, contributing to their erythrocytes’ redox status imbalances. We studied 15 control and 24 obese prepubertal children, 12 of whom were insulin-resistant according to an oral glucose tolerance test (OGTT). We analyzed erythroid malondialdehyde (MDA) and carbonyl group levels as oxidative stress markers. NADP+/NADPH and GSH/GSSG were measured to determine redox status, and NADPH production by both G6PDH and 6PGDH was assayed spectrophotometrically to characterize pentose phosphate pathway activity. Finally, superoxide dismutase (SOD), catalase (CAT), glutathione peroxidase (GPX) and glutathione reductase (GR) activities were also assessed. As expected, MDA and carbonyl groups levels were higher at baseline and along the OGTT in insulin-resistant children. Both redox indicators showed an imbalance in favor of the oxidized forms along the OGTT in the insulin-resistant obese group. Additionally, the NADPH synthesis, as well as GR activity, were decreased. H_2_O_2_ removing enzyme activities were depleted at baseline in both obese groups, although after sugar intake only metabolically healthy obese participants were able to maintain their catalase activity. No change was detected in SOD activity between groups. Our results show that obese children with insulin resistance present higher levels of oxidative damage, blunted capacity to generate reducing power, and hampered function of key NADPH-dependent antioxidant enzymes.

## 1. Introduction

Worldwide, more than 300 million children and teenagers between the ages of 5 and 19 years and 41 million children under the age of 5 were overweight or obese in 2016 according to the WHO’s reports [1,2]. Obesity is defined as excessive adiposity caused by energetic imbalance. Excessive fat accumulation is related to chronic inflammation. Multiple factors contribute to this relation—innate immune response activation, changes in cytokines secretion (IL-6, TNF-a, MCP-1, adiponectin and leptin), tissue oxygen depletion, cell necrosis, and lipid metabolism dysregulation have all been implicated [3,4]. Proinflammatory events are elicited in a context of oxidative stress due to reactive oxygen species (ROS) overproduction. Oxidative stress plays a physiological role by inducing adaptive responses (phenomenon known as oxidative eustress), but it may also lead to damage (oxidative distress) [5]. Accordingly, lipid peroxidation products may trigger both cellular adaptive responses and cytotoxicity [6]. Cell tolerance to oxidative stress is primarily enhanced through induction of antioxidant enzymes which may be compromised as the disease evolves, switching from a eustress to a distress situation [7]. Oxidative stress plays a pivotal role in the development of a wide variety of diseases, such as diabetes, certain types of cancers, as well as neurodegenerative and cardiovascular diseases [8,9,10,11,12].

Erythrocytes are very particular cells. They are ubiquitous, have a relatively high turnover rate, lack nuclei, mitochondria and other organelles, and are extremely specialized. Due to their main function as oxygen transporters and their high iron content, erythrocytes are highly exposed to reactive oxygen/nitrogen species (ROS/RNS), and also to external inputs such as advanced glycation/lipoxidation end products (AGEs/ALEs) [13,14,15]. Thus, they are equipped with powerful antioxidant systems which may be classified into enzymatic (catalase, superoxide dismutase, thioredoxin, etc.) and nonenzymatic defenses (ascorbic acid, glutathione, etc.) responsible for controlling redox balance [16,17,18]. This makes erythrocytes reflect variations in oxidative status very early and have been proposed as sensors of oxidative stress [16]. In fact, a group of obese children with metabolic complications (insulin resistance, IR) presented antioxidant response depletion and enhanced oxidative stress markers in response to an oral glucose tolerance test (OGTT), while metabolically healthy obese children were able to effectively increase their antioxidants defenses to avoid oxidative damage [16]. Some of the above-mentioned antioxidant defenses need to be supplied with reducing power to exert their function. Glutathione, for example, needs to be recycled (when oxidized) by glutathione reductase, an NADPH-dependent enzyme, and catalase is also NADPH-dependent to avoid inactivation [19]. Moreover, NADPH oxidase or inducible nitric oxide synthase (eNOS) activities are dependent on NADPH in the erythrocyte [20].

Under physiological conditions, most of the glucose consumed by the erythrocyte is derived from the production of energy by glycolytic activities. Only a small fraction is used in the pentose phosphate pathway (PPP) to provide reducing power in the erythrocyte. In the PPP, glucose-6-phosohate dehydrogenase (G6PDH) and 6-phosphogluconate dehydrogenase (6PGDH) catalyze the reduction of NADP^+^ to NADPH, renewing its antioxidant power. To this end, they use the energy resulting from glucose-6-phosphate conversion to 6-phosphogluconolactone and 6-phosphogluconate to ribulose-5-phosphate, respectively. Under oxidative stress, this cycle must be upregulated to increase its activity, which can be as much as 20-fold. In this sense, the first enzyme in the pathway (G6PDH) plays a pivotal role in its regulation, since its activity is controlled by the NADP^+^/NADPH ratio [21,22]. The potential role of PPP dysregulation in the oxidative damage associated with obesity and its comorbidities remains unexplored.

In this work, we aim to assess the potential role of the PPP by analyzing the activity of the main NADP dehydrogenases and their ability to provide reducing power in the erythrocytes of obese children with insulin resistance (ObIR+) as compared to a group of metabolically healthy obese (ObIR−) participants and a group of healthy controls. Our data confirm the presence of an oxidative environment in a population of prepuberal ObIR+ children, and evidence in these a dysregulated PPP activation, resulting in a deficient response to a glucose overload as compared with the other study groups.

## 2. Material and Methods

### 2.1. Subjects

Obese children requiring an OGTT to assess their carbohydrate metabolism were recruited at our Pediatric Endocrinology Unit (*n* = 24) in Puerta del Mar University Hospital, Cádiz, Spain. Nonobese children who needed a blood test (generally for preanesthesia), were recruited as control group (*n* = 15). All of them were prepubertal children, between 6 and 10 years of age.

Inclusion criteria for the obese group were children between 6 and 10 years in a tanner stage I with a BMI > +2 SD and, as mentioned above, with a medical prescription of an OGTT. On the other hand, the control group had a BMI < +2 SD and, as the obese group, an age between 6 and 10 years and Tanner I. We subdivided the obese group according to the result of the OGTT and their basal glucose and insulin levels. Patients were classified into the ObIR− group when they showed no evidence of carbohydrate metabolic impairment and into the ObIR+ group when presenting at least one of the following criteria: Impaired fasting glucose (IFG), impaired glucose tolerance (IGT) or insulin resistance (IR), according to the ADA criteria (a homeostasis model assessment of insulin resistance (HOMA-IR) score above 3.5, or fasting insulin > 15 µUI/mL, insulin at 120 min of OGTT > 75 µUI/mL or insulin at any time point of the curve > 150 µUI/mL [23]). Every OGTT was performed by a trained nurse in the clinic, after overnight fasting, with 1.75 g/kg (maximum 75 g) of sucrose (GlycoSull, QCA S.A., Tarragona, Spain). The control group was not subjected to the OGTT.

### 2.2. Sample Collection and Preparation

Venous blood samples were extracted in serum (gelose) and plasma (EDTA) separating tubes from every child after overnight fast (T0). For obese children, blood samples were also collected at 60 and 120 min during the OGTT. Every tube was processed as previously described [9]. Both blood collection tubes were centrifuged at 1500× *g* for 10 min at 4 °C to obtain serum and plasma samples. Plasma-separating tubes were also used for the immediate purification of red blood cells (RBCs) for determination of oxidative stress and antioxidant biomarkers. Briefly, erythrocytes were washed 3 times with 5 mL of cold saline solution (NaCl 9 g/L) to obtain the RBC fraction by centrifuging for 5 min at 1500× *g* and 4 °C. In total, 300 µL of this RBC solution was lysated by hypotonic shock with 4 mL of distilled water, and then subjected to centrifugation at 17,000× *g* for 10 min at 4 °C to separate membranes (pellet) and cytosol (supernatant). The pellet was resuspended in RIPA buffer, while the cytosolic fraction was treated with 750 µL Chloroform:Ethanol (1:2; *v*/*v*) to remove hemoglobin. To obtain hemoglobin-free cytosolic fractions, samples were centrifuged again at 1500× *g* for 10 min at 4 °C. Every fraction was immediately frozen in liquid nitrogen to ensure its correct preservation.

### 2.3. Anthropometry and Biochemical Analysis of the Study Population

Demographic data, personal and familiar cardiovascular antecedents, and risk factors were collected from the electronic medical records. Anthropometric data were measured and puberal findings were evaluated by pediatric endocrinologists. Z-scores for age and gender for every anthropometric variable were also registered using Spanish reference values.

Biochemical analysis included insulin and glucose determination at basal and at different times along the OGTT. The homeostasis model assessment of insulin resistance (HOMA-IR) was also calculated by applying the formula: HOMA-IR = [(fasting insulin (mIU/L) × fasting glucose (mg/dL) × 0.055/22.5]. Biochemical analysis was performed on serum by using standard clinical assays at the Clinical Analysis Department of our Hospital. Insulin was measured in a Roche Analytics E170 analyzer using the “INSULIN 100 TESTS/KT” (Roche Diagnostics, Indianapolis, IN, USA) (imprecision insulin assay was 2.26%), and serum glucose was analyzed by the hexokinase method in a C8000 analyzer (Roche Diagnostics, Indianapolis, IN, USA) (imprecision glucose assay was 2% at standard level (100 mg/dL) and 3% at top level (240 mg/dL)).

### 2.4. Lipid Peroxidation Quantification

The formation of thiobarbituric acid-reacting substances (TBARS) was measured according to the method described by Buege et al. [24]. In total, 100 µL of diluted RBC samples (1:1, *v*/*v*; in water) was mixed with 400 µL of the reaction solution, composed by 2.5 N HCl, 0.375% (*w*/*v*) thiobarbituric acid (TBA), 15% (*w*/*v*) trichloroacetic acid, and 0.01% (*w*/*v*) butylated hidroxytholuene. Then, for lipid fraction purification and incubation with TBA, the mix was heated at 100 °C for 15 min and, after cooling, centrifuged at 900× *g* for 5 min. The malondialdehyde (MDA) standard curve was prepared in the same way but without heating and centrifuging. Finally, 200 µL of each sample and MDA curve were added to a 96-well microplate and the absorbance was recorded at 535 nm. Results were expressed as nmol of MDA per mg of proteins in RBCs.

### 2.5. Carbonyl Groups Determination

For quantifying oxidative modification of proteins, carbonyl groups (C = O) were determined following the method described by Levine et al. [25]. It was based on the purification of proteins from samples and their incubation with 2,4-dinitrophenylhydrazine (DNPH) in 6 M HCl-guanidine, which when reacting with the carbonyl groups produce 2,4-phenylhydrazone. In the presence of 10 M KOH, hydrazone can be determined spectrophotometrically by its absorbance at 370 nm. Each sample had its own blank (without DNPH). Differences in absorbance between samples and blanks were employed to quantify the content of carbonyl groups. Absorbance at 280 nm measured in blanks was used to estimate the protein content of the samples using a BSA standard curve prepared in 6 M HCl-guanidine. Results are expressed as nmol of C = O per mg of proteins in RBCs.

### 2.6. Glutathione Availability

The analysis of RBC glutathione equivalents (tGSH) was performed following the “DTNB-GSSG Reductase Recycling Assay for GSH and GSSG”, based on the GSH oxidation to GSSG by the 5,5′-dithiobis-2-nitrobenzoic acid (DTNB) [26]. The product, 5-thio-2-nitrobenzoic acid (TNB), can be monitored by spectrophotometry at 412 nm. Since GSSG was reduced to GSH, the sum of GSH and GSSG (tGSH) could be measured when a highly specific glutathione reductase and NADPH was present in the reaction. GSSG concentration can be analyzed by the same method if samples are previously derivatized with Triethanolamine (TEA) and 2-vinylpyridine. Previous to this analysis, samples were diluted (1:5) in 5% metaphosphoric acid and centrifugated for the elimination of proteins’ sulfhydryl groups. Data were expressed as nmol of GSH/GSSG/tGSH per mg of protein. Finally, redox status was expressed as GSH/GSSG.

### 2.7. NADPH Availability

Total NADP (tNADP) and NADPH were quantified in erythrocytes using the commercial kit “NADP/NADPH Assay kit”, following the manufacturer’s instructions (Abcam, Cambridge CB4 OFL, UK). Briefly, erythrocytes were incubated at 60 °C for 30 min to decompose NADP^+^. Then, 10 µL of treated and untreated samples were placed in a 96-well plate for the spectrophotometric determination of NADPH and NADP^+^ concentrations, respectively, at 450 nm. This technique enables the extrapolation of NADP^+^ concentration in order to estimate the NADP^+^/NADPH ratio as a redox status indicator. Data are expressed as pmol of NADP^+^ or NADPH per mg of protein.

### 2.8. NADP-Dehydrogenase Activities

Both G6PDH (EC:1.1.1.49) and 6PGDH (EC:1.1.1.44) activities were determined spectrophotometrically by quantifying the rate of NADP^+^ reduction as described by Mateos et al., 2009 [27]. Therefore, 10 µL of sample was mixed in the wells of a microplate with 36.11 mM HEPES and 0.8 mM NADP^+^. Finally, substrate (glucose-6-phosphate for G6PDH activity and 6-phosphogluconate for 6PGDH activity) was added to start the reaction. Absorbance was followed at 340 nm for 3 min with determinations every 20 s. The molar extinction coefficient for NADP^+^ was 6.22 mM^−1^ cm^−1^. The enzymatic activity was expressed as nmol NADP min^−1^ per mg of protein.

### 2.9. Glutathione Reductase Activity

Glutathione reductase (EC 1.8.1.7) activity was determined by following the NADPH oxidation rate that the enzyme uses to recycle reduced glutathione (GSH) from oxidized glutathione (GSSG), as described by Edwards et al. [28]. In total, 0.1 M HEPES-NaOH buffer pH 7.8 with 1 mM EDTA and 3 mM MgCl_2_ was added to a 96-well microplate together with 0.5 mM GSSG (samples) or without it (blanks). Then, 5 µL of sample and 0.2 mM NADPH were added to start the reaction. Absorbance was followed at 340 nm for 2 min with determinations each 10 s. The molar extinction coefficient for NADPH was 6.22 mM^−1^ cm^−1^. The enzymatic activity was expressed as nmol NADP min^−1^ per mg of protein.

### 2.10. Glutathione Peroxidase Activity

Glutathione peroxidase (EC 1.11.1.9) activity was measured spectrophotometrically using the “Glutathione Peroxidase Assay Kit” (Abcam, Cambridge CB4 OFL, UK), following manufacturer’s instructions. Glutathione peroxidase (GPX) catalyzes the reduction of H_2_O_2_ to H_2_O via GSH oxidation to GSSG. The decrease in NADPH, proportional to GPX activity, was monitored at 340 nm. The enzymatic activity was expressed as nmol H_2_O_2_ min^−1^ per mg of protein.

### 2.11. Catalase Activity

Catalase (EC 1.11.1.6) activity was measured following the protocol described by Aebi et al. [29], based on the absorbance decrease at 240 nm due to the catalase mediated H_2_O_2_ decomposition. Briefly, samples were diluted with distilled water (1:100 *v*/*v*), and 2 µL of the diluted samples was added to 50 mM phosphate buffer at pH 7 with 9.96 mM H_2_O_2_. The absorbance was measured at 240 nm. The enzymatic activity was expressed as nmol H_2_O_2_ min^−1^ per mg of protein, with a molar extinction coefficient for H_2_O_2_ of 39.58 M^−1^ cm^−1^.

### 2.12. Superoxide Dismutase Activity

Superoxide dismutase (EC 1.15.1.1) activity was measured in erythrocytes using the commercial kit “EpiQuik™ Superoxide Dismutase Activity/Inhibition Assay Kit”, following manufacturer’s instructions (Epigentek, Farmingdale, NY, USA). The principle of this assay is based in the formation of a colored water-soluble formazan upon reduction of a dye with superoxide anion. Thus, the higher the superoxide dismutase (SOD) activity is in the sample, the lower the rate formation of formazan that takes place, resulting in lower OD values at 470 nm. Data are shown in mU/µL*mg protein.

### 2.13. Statistical Analysis

The significance tests used were the nonparametric test: the Mann–Whitney U test for pairwise comparisons, and Kruskal–Wallis test (followed by the Dunn’s test) for comparisons of more than two groups. When comparing categorical variables, we applied the Chi-square statistic. The sample size was calculated using GRANMO 7.12 sample size calculator open software [30], based on previous data on lipid peroxidation levels [16]. Hence, accepting an alpha risk of 0.05 and a beta risk of 0.2, assuming a common standard deviation of 0.47, the estimated sample size to recognize as statistically significant a difference greater or equal to 0.6 units is 10 subjects per group, for the comparisons between groups. For intragroup comparisons (repeated measurements), and accepting the same risks in a two-sided test, 8 subjects per group were calculated as necessary to recognize statistically significance, which was determined as a difference greater than 0.46, assuming a common standard deviation of 0.5 and a correlation coefficient between initial and final measurements of 0.8. Eventually, the statistical power of our sample was calculated, considering the real data of each variable, and obtained a mean power of 81.29%.

## 3. Results

### 3.1. Anthropometry and Biochemical Analysis of the Study Population

As shown in Table 1, age was similar in every group. Distribution according to sex was similar between controls and ObIR−, but in the ObIR+ group there were twice as many boys than girls. There were no clinically relevant differences between groups in any of the neonatal variables. ObIR+ had lower mean weight at birth than ObIR−, but all three groups had mean weights at birth within normal values (between −2 and +2 SD). In addition to the obvious differences in obesity-related parameters, such as waist circumference and BMI (SD), between nonobese and obese children, no other differences were found between ObIR− and ObIR+ anthropometric variables. Similarly, blood pressure was higher in both obese groups as compared to controls, but there were no differences between obese groups with or without IR.

As expected by the definition of the study groups, there were significantly higher insulin levels in the OGTTs and elevated HOMA-IR indexes in the ObIR+ with respect to control and ObIR− groups. Interestingly, basal insulin and HOMA-IR index was also higher in the ObIR− group than in controls, although both levels were within the normal ranges. Glucose levels, however, were similar between controls and ObIR−, whilst ObRI+ had slightly higher basal glucose, still within normal levels. Both obese groups had similar TGs, and these were approximately double those in the control groups. Total and LDL-cholesterol values were not different among any group, while HDL-cholesterol was lower in both obese populations, without differences between ObIR− and ObIR+. Finally, ferritin levels in the ObIR+ were twice as high as in any other group, and this difference was statistically significant. Creatinine levels were also higher in ObIR+ groups when compared to controls.

### 3.2. Oxidative Damage

**Figure 1:**
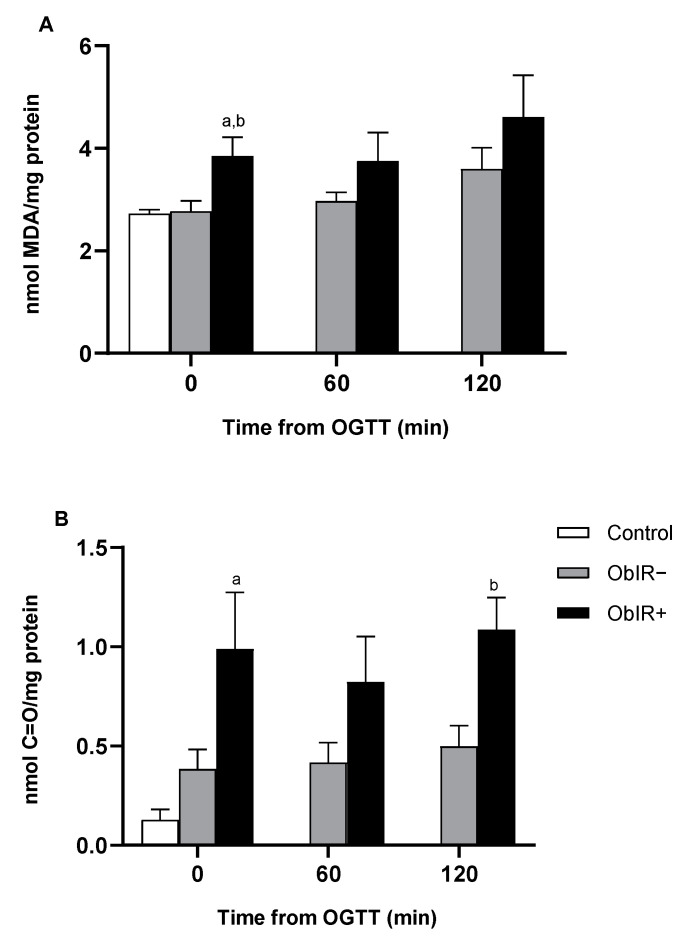
Levels of oxidative damage in red blood cells (RBCs) of healthy and obese children. Malondialdehyde (MDA) (**A**) and carbonyl group levels (**B**) were measured in RBCs at baseline and along oral glucose tolerance test (OGTT). White bars represent the control group, grey bars represent ObIR− group, and black bars represent obese children with insulin resistance (ObIR+) group. Values are means ± SEM. *p* < 0.05 was considered for statistical significance. (a) shows significant differences relative to control at baseline and (b) shows significant differences relative to ObIR−.

MDA levels were analyzed in total RBC samples, whilst carbonyl groups were only determined in the RBC intracellular fraction. We found baseline levels of MDA to be higher in the ObIR+ group when compared to the control group. Additionally, MDA values showed tendency to increase along the OGTT in the ObIR+ group, although no significant differences were found in any other study point (Figure 1A). Carbonyl groups profile showed a 5-fold increase in the ObIR+ group with respect to the control group at baseline, and 2-fold increase over the ObIR− group at 120 min after OGTT (Figure 1B).

### 3.3. Glutathione Availability

**Figure 2:**
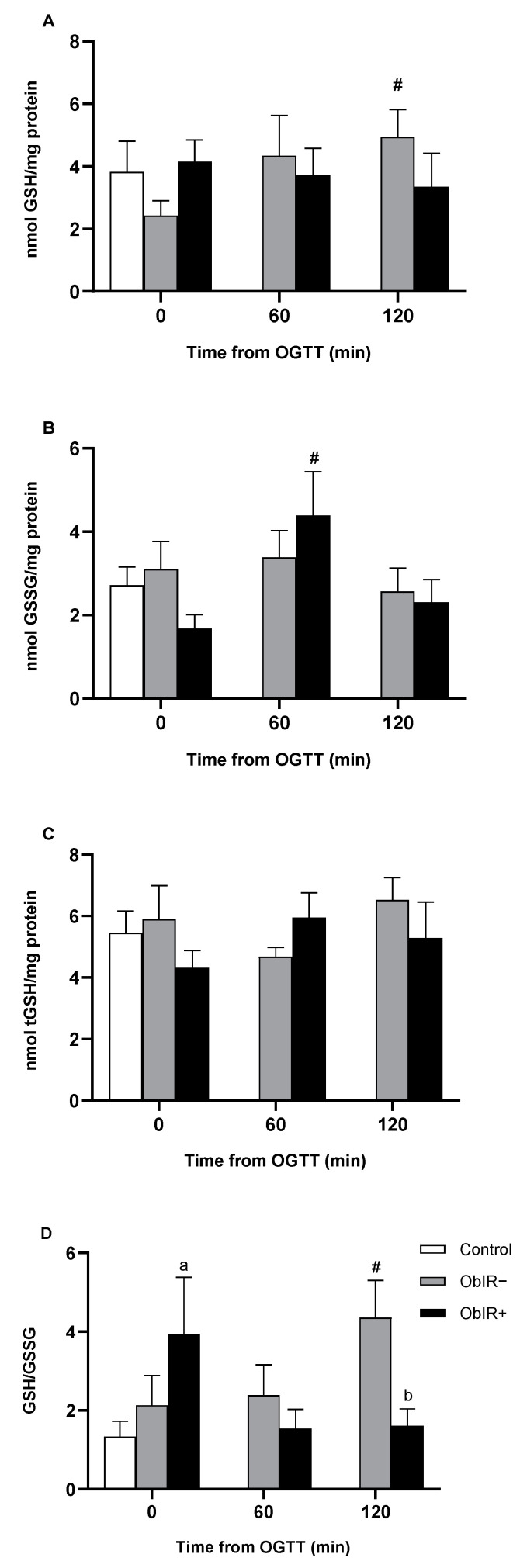
Glutathione availability in RBCs of healthy and obese children. GSH (**A**) and GSSG (**B**) were measured in RBCs at baseline and along OGTT. tGSH (**C**) and GSH/GSSG (**D**) were also calculated. White bars represent the control group, grey bars represent ObIR− group, and black bars represent ObIR+ group. Values are means ± SEM. *p* < 0.05 was considered for statistical significance. (a) shows significant differences relative to control at baseline, (b) shows significant differences relative to ObIR−, and (**#**) shows intragroup differences along the OGTT.

ObIR+ children exhibited an increase in GSSG levels 60 min after the OGTT; in contrast, only ObIR− experienced changes in GSH values along the curve, reaching the maximum at the 120 min time point (Figure 2A,B). No changes were found in tGSH levels in both obese groups along the OGTT (Figure 2C). Finally, GSH/GSSG was disbalanced at baseline in ObIR+ and it changed after an OGTT in both obese group, resulting in a decrease along the curve in ObIR+, with significant changes at 120 min, whilst ObIR− increased GSH/GSSG along the curve (Figure 2D).

### 3.4. NADPH Availability

**Figure 3:**
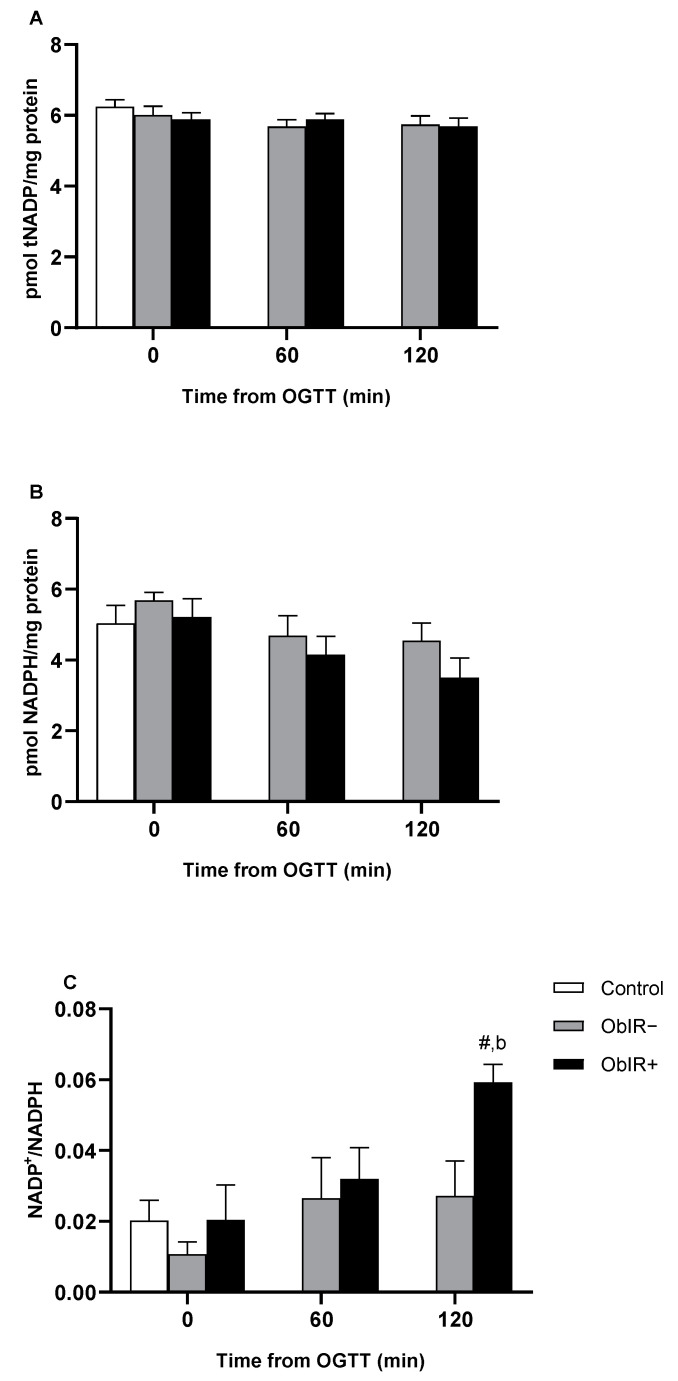
NADPH availability in RBCs of controls and obese children. Total NADP (tNADP) (**A**) and NADPH (**B**) were measured in RBCs at baseline and along OGTT. NADP^+^ data were extrapolated from both total NADP and NADPH and used to calculate the NADP^+^/NADPH ratio (**C**). White bars represent the control group, grey bars represent ObIR− group, and black bars represent ObIR+ group. Values are means ± SEM. *p* < 0.05 was considered for statistical significance. (b) shows significant differences relative to ObIR−, and (**#**) shows intragroup differences along the OGTT.

Total NADP levels were similar in all three groups at baseline and remained unchanged along the OGTT in both obese groups (Figure 3A). Levels of NADPH were also similar in all three groups at baseline and remained in similar values in ObIR− along the curve, while the ObIR+ group showed a decreasing trend that did not reach statistical significance (*p* = 0.098) (Figure 3B). Conversely, NADP^+^/NADPH ratio was similar between groups at baseline, but markedly increased at 120 min of the OGTT only in the ObIR+ obese group (Figure 3C).

### 3.5. NADP-Dehydrogenases Activities

**Figure 4:**
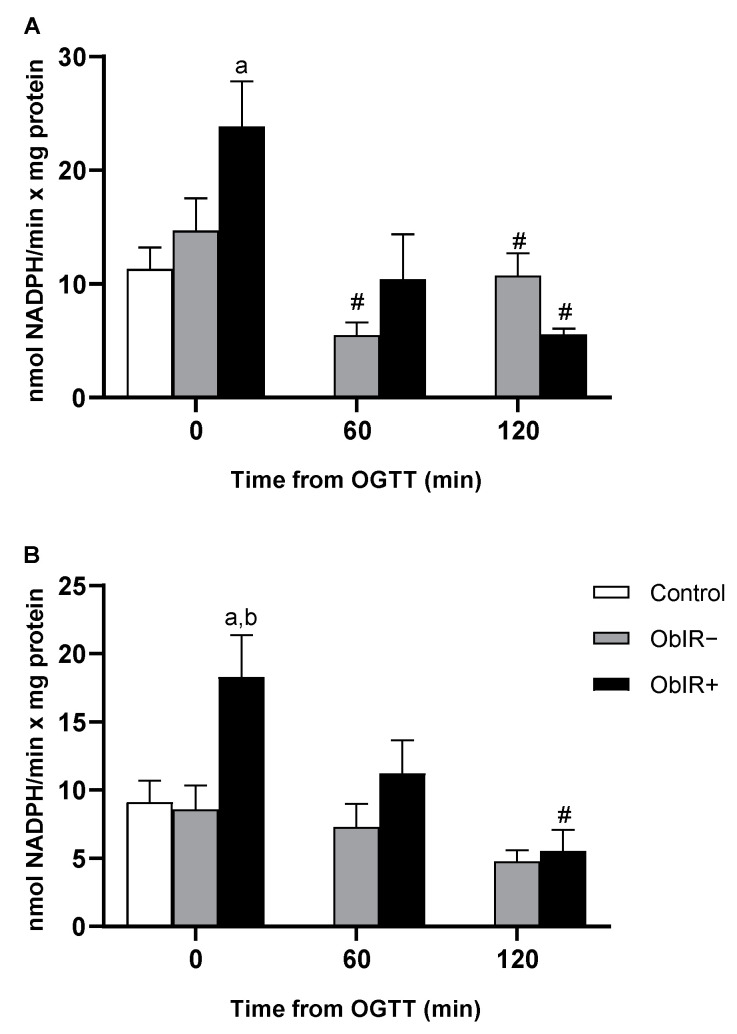
NADP^+^-dehydrogenase Activity in RBCs of healthy and obese children. Glucose-6-phosphate dehydrogenase (**A**) and 6-phosphogluconate dehydrogenase (**B**) activities were measured in RBCs at baseline and along OGTT. White bars represent the control group, grey bars represent ObIR− group, and black bars represent ObIR+ group. Values are means ± SEM. *p* < 0.05 was considered for statistical significance. (a) shows significant differences relative to control at baseline, (b) shows significant differences relative to ObIR−, and (**#**) shows intragroup differences along the OGTT.

Both G6PDH and 6PGDH activities were quantified in RBCs. The activities of both dehydrogenases appeared to be exacerbated at baseline in ObIR+, with significant differences when compared to controls in G6PDH and to control and ObIR− in 6PGDH. However, along the curve, both G6PDH and 6PGDH activities suffered a marked decrease in ObIR+. On the other hand, the ObIR− group decrease their G6PDH activity at 60 min, which was recovered to basal levels at 120 min (Figure 4A,B).

### 3.6. Glutathione Reductase Activity

**Figure 5:**
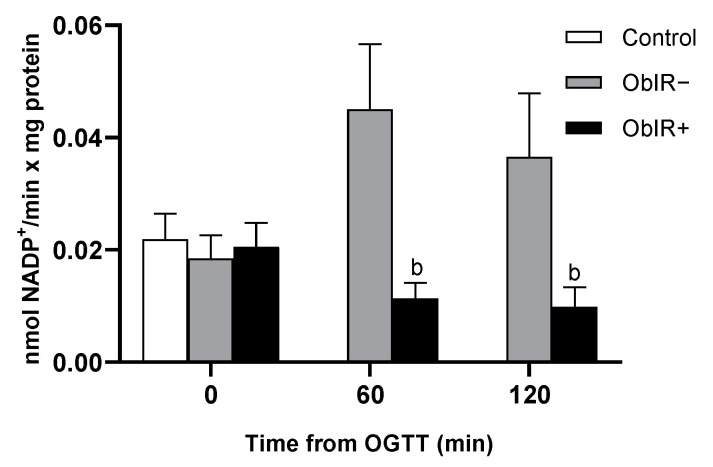
Glutathione reductase activity in RBCs of healthy and obese children. Glutathione reductase activity were measured in RBCs at baseline and along OGTT. White bars represent the control group, grey bars represent ObIR− group, and black bars represent ObIR+ group. Values are means ± SEM. *p* < 0.05 was considered for statistical significance. (b) shows significant differences relative to ObIR−.

Glutathione reductase (GR) activity, measured in RBC cytoplasm, peaked in ObIR− at 60 min, while ObIR+ did not experience any change along the curve, with significant differences between both obese groups 60 and 120 min after the OGTT (Figure 5).

### 3.7. H_2_O_2_ Removing Enzymes

**Figure 6:**
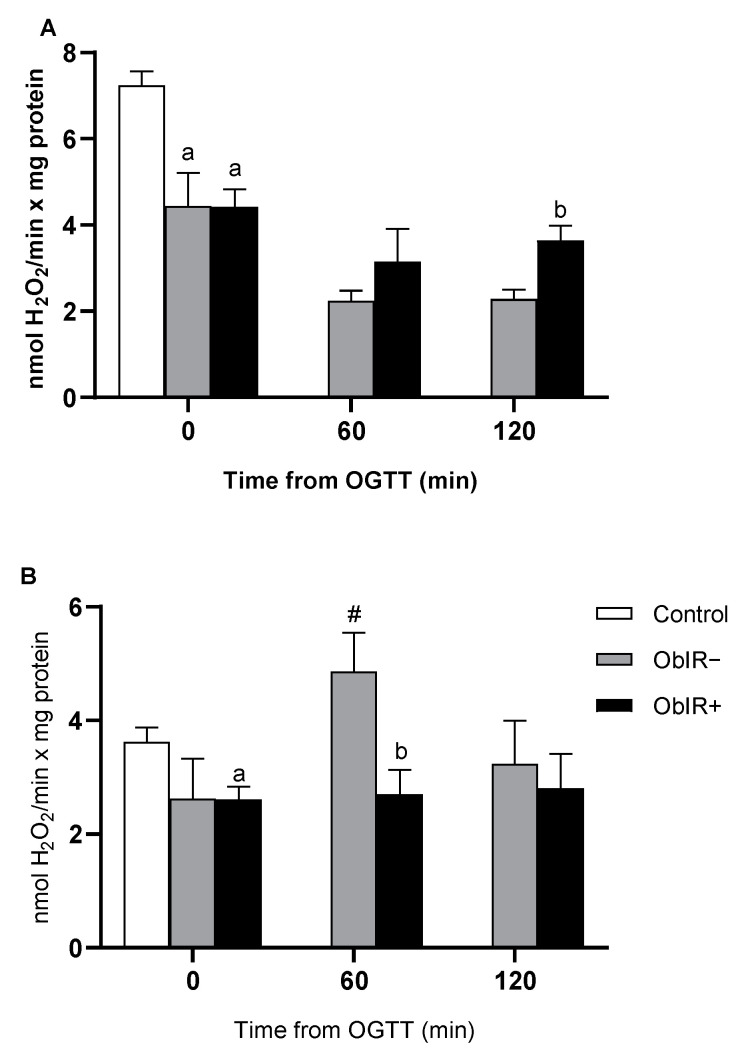
H_2_O_2_ removing enzymes. Glutathione peroxidase (**A**) and catalase (**B**) were measured in RBCs of healthy and obese children at baseline and along OGTT. White bars represent the control group, grey bars represent ObIR− group, and black bars represent ObIR+ group. Values are means ± SEM. *p* < 0.05 was considered for statistical significance. (a) shows significant differences relative to control at baseline, (b) shows significant differences relative to ObIR−, and (**#**) shows intragroup differences along the OGTT with respect to baseline values.

Glutathione peroxidase (GPX) and catalase (CAT) activities were decreased at baseline in both obese groups as compared to controls (Figure 6A,B, respectively), although only the ObIR+ group reached statistical difference in their catalase activity at this point. GPX activity did not increase along the curve in neither obese group. Additionally, whilst ObIR+ were unable to increase levels of catalase activity along the curve, ObIR− responded to the OGTT increasing their catalase activities at 60 min.

### 3.8. Superoxide Dismutase Activity

**Figure 7:**
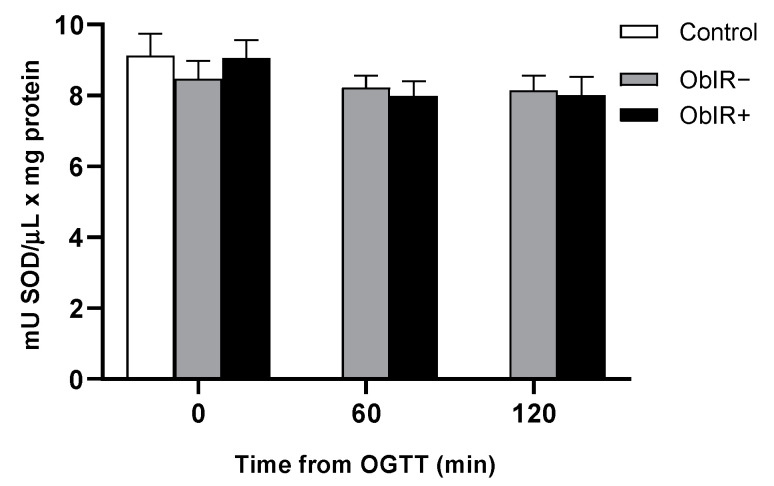
Superoxide dismutase activity were measured in RBC of healthy and obese children at baseline and along OGTT. White bars represent the control group, grey bars represent ObIR− group, and black bars represent ObIR+ group. Values are means ± SEM. *p* < 0.05 was considered for statistical significance.

Superoxide dismutase (SOD) activity was similar at baseline and remained unchanged along the OGTT in every group (Figure 7).

A graphic summary of our results is shown in the Supplementary Figure S1.

## 4. Discussion

Obesity and childhood obesity have become one of the most relevant diseases from the end of last century since their prevalence have increased in the past decades at exponential levels. Their incidence, together with the associated comorbidities and risk of developing other diseases in the future, make obesity an issue of major importance. Oxidative stress has been widely related to the pathophysiologies of several disorders and associated with childhood obesity [16,31]. Since oxidative stress-mediated complications may play a pivotal role in the development of obesity-related comorbidities a special effort must be made to assess the biological and molecular bases of this oxidative dysfunction.

In this work, we describe MDA and carbonyl groups to be elevated in ObIR+ children’s erythrocytes when compared with a control group at baseline and compared to ObIR− children after an OGTT. These results reproduce previously published data in a cohort of children between 4 and 14 years [16], reinforcing the evidence of the relation between metabolic impairment and oxidative stress even at pediatric ages, although in the present study, this is more specifically before puberty. Elevated concentrations of lipid peroxidation markers in urine samples of obese children have been described to be predictors of higher risk of developing cardiovascular diseases [32], while carbonyl groups have also been related to childhood obesity and metabolic syndrome in several works [33,34].

Erythrocytes are circulating cells exposed to the action of endogenous and exogenous sources of ROS and, therefore, targets of systemic oxidative stress. ROS generated by other cells in the circulatory system (endothelial cells, monocytes or neutrophils) may penetrate into the erythrocyte [35]. Furthermore, endogenously, hemoglobin autoxidation, NADPH oxidase activity or the Fenton and Haber–Weiss reactions contribute to the generation of ROS within the erythrocyte, as potential sources of oxidative stress [36]. Therefore, the availability of reducing power (NADPH) to control glutathione homeostasis in these cells, as well as the activity of the antioxidant enzymes involved in glutathione metabolism, is essential to maintain the physiological response of the cell to these potential oxidative stress sources to which it is exposed.

To estimate the redox status in red blood cells, glutathione and NADPH availability were assessed. Both redox indicators were imbalanced in favor of the oxidized forms in those obese children with IR. On the contrary, GSH/GSSG in ObIR− increased in response to sugar intake. Our previous work also found an altered GSH/GSSG ratio in ObIR+ children, whilst the ObIR− group was able to increase the amount of GSH over the oxidized form [16]. These results show how IR induces redox impairments even in prepuberal children, stressing the importance of controlling metabolic dysregulation from early ages. In line with these findings, cells with lower GSH levels have been shown to be more sensitive to oxidative damage [37]. Moreover, altered glutathione homeostasis has been related to several diseases such as tuberculosis, human immunodeficiency virus (HIV) infection, diabetes, Parkinson’s disease or cardiovascular diseases [38,39,40].

Considering the inability of insulin-resistant obese children to correctly recycle the oxidized form of the endogenous antioxidant glutathione to its reduced form under stressful conditions, we evaluated the behavior of the antioxidant enzyme glutathione reductase, responsible for the reduction of GSSG into GSH, with the consumption of reducing power in form of NADPH. Although controls and both obese groups had similar glutathione reductase activity levels at baseline, in response to the OGTT only the metabolically healthy obese group was able to increase the glutathione reductase activity. This result could explain, at least in part, the differential dynamics observed in glutathione availability between groups, since ObIR− effectively increased GSH concentration along the curve, whilst ObIR+ experienced an increase in GSSG. Glutathione reductase activity has been previously described to be decreased in childhood obesity and in metabolic syndrome. Moreover, in an interventional study in a group of obese women, weight loss led to an increase in glutathione reductase activity [41,42,43]. On the other hand, some authors found morbidly obese adults, and adults with metabolic syndrome, to have increased levels of glutathione reductase activity and even glutathione reductase protein levels [44,45,46]. To the best of our knowledge, the role of insulin resistance in this antioxidant enzyme in obese children has not yet been looked at to date. Our results establish a link between altered glutathione reductase activity and the pathophysiology of childhood obesity metabolic complications.

Since glutathione reductase needs NADPH to accomplish the recycling of GSSG into GSH, any disturbance in the PPP may eventually lead to altered glutathione reductase activity and thus, inefficient antioxidant defenses. As described above, although ObIR+ children had exacerbated G6PDH and 6PGDH activities at baseline, an acute glucose intake resulted in a marked decrease in both enzymes’ activities, depriving the erythrocyte of the necessary reducing power to face the generated stress. Higher baseline levels of G6PDH and 6PGDH activities may trigger deleterious signaling effects, since the NADPH generated is a substrate for the superoxide producer NADPH oxidase (NOX). In fact, NOX and the inducible nitric oxide synthase (iNOS) have been described as playing a key role in ROS generation in obese adipose tissue that may lead to insulin resistance. Moreover, upregulated G6PDH levels/activity were observed in many cancer types [47]. In line with this, erastin-mediated ferroptosis (programmed cell death dependent on iron) was lowered by NOX/PPP inhibitors and in knockout mice of two PPP enzymes, G6PDH and 6PGDH, suggesting an implication of NOX and some PPP enzymes in this newly described form of regulated cell death characterized by reduced GSH levels and increased lipid peroxidation [48]. Thus, NADPH has a dual role in ROS generation. In line with this, RBCs are highly dependent on G6PDH-derived NADPH to maintain the balance between the oxidized and reduced forms of glutathione. The lack in erythrocytes of any other reducing power source, makes them extremely sensitive to oxidative stress under impaired G6PDH activity conditions [49]. Additionally, in studies of animal models (high fat diet (HFD) mice), skeletal muscle G6PDH has been shown to be defective under insulin-stimulated conditions, in line with our results [50]. High glucose concentration-mediated impaired G6PDH activity has also been related to an increase in ROS production and reduction in cell survival in endothelial and kidney cells [51].

The decrease in NADPH availability after the OGTT in ObIR+ is related to an impaired activity of NADPH-dependent enzymes such as glutathione peroxidase and glutathione reductase. Additionally, catalase remains unable to increase response to OGTT in ObIR+, as previously described [16]. Although catalase activity does not strictly depend on NADPH, it has been described that NADPH intervenes in the stabilization of the active form of the protein [19]. We now reproduce this result in a cohort of prepuberal children, clarifying the importance of a continuous reducing power supply in the defense against stressful stimuli. This has been previously shown in mice lacking catalase, which develop obesity and a prediabetic phenotype [52]. Rupérez et al. found catalase activity to be negatively correlated with childhood obesity and insulin resistance [53]. As commented above, increased baseline G6PDH or 6PGDH activities may lead to a rise in superoxide production via NOX. Superoxide should be converted into H_2_O_2_ by the superoxide dismutase, but according to our data, superoxide dismutase activity is similar between groups at baseline and remains unchanged between obese groups along the curve, probably because of the deviation of superoxide radicals to Fenton and Haber–Weiss reactions (Supplementary Figure S1). Indeed, plasma ferritin levels were considerably increased in ObIR+ with respect to controls and metabolically healthy obese children. Increased H_2_O_2_ concentrations, in turn, may also affect catalase activity, since H_2_O_2_ has been shown to produce reversible inhibitions and irreversible inactivation to bovine catalase [54,55]. Furthermore, S-Nitrosation has been found to mediate catalase activity loss in gestational diabetes mellitus, postulating that ROS/RNS-mediated post-translational modifications of antioxidant enzymes such as catalase must be considered in metabolically unhealthy childhood obesity-related oxidative stress [56]. Indeed, we have recently provided some evidence of catalase S-Nitrosation in obese children [57]. The increase in superoxide and NO generates ONOO^−^, which may also mediate the nitration of catalase and other enzymes in the erythrocyte, such as hemoglobin, which can be a marker of nitro-oxidative stress [58,59].

One limitation of our study is the relatively small group size which suggests our results should be interpreted with caution. It would be desirable to scale-up the sample size in the near future, including patients from different centers and different origins. This highlights another limitation of our study, which is the use of classical (though well validated), techniques to perform our measurements, requiring immediate and complex processing of blood samples. This limits the possibility to receive samples from different centers to be analyzed. Thus, it would be desirable to replicate these results with novel methodological strategies to facilitate reproducibility and to perform multicenter studies.

## 5. Conclusions

Altogether, our data suggest that the availability of NADPH in compromised situations of metabolic stress, induced by glucose intake, plays a pivotal role in the antioxidant response of erythrocytes. We have found a relation between erythrocytes’ altered reducing power supplies and the oxidative stress-mediated pathophysiology of insulin resistance in prepubertal obese children. The inability of ObIR+ children’s erythrocytes to synthesize or recycle NADPH due to blunted dehydrogenases activities leads to an altered redox status, as shown by lowered GSH levels, since glutathione reductase lacks the reducing power needed for this process. In this situation, many antioxidant enzymes may not fully exert their functions to neutralize oxidizing species generated in the red blood cells by multiple mechanisms. Excess reactive species lead to lipid and protein oxidation which, in turn, may affect the integrity of the erythrocyte.

We found a physiological deviation of redox balance (oxidative eustress) in obese children without insulin resistance, and a supraphysiological deviation (oxidative distress) in the insulin-resistant group.

## Figures and Tables

**Table 1:** Anthropometric and biochemical characteristics of the study population.

	Control	ObIR−	ObIR+
Anthropometric parameters			
Number of subjects (N)	15	14	10
Sex	8♂ 7♀	6 ♂ 8 ♀	7♂ 3 ♀
Age (years)	8.33 ± 0.42	8.79 ± 0.48	8.71 ± 0.44
WC (cm)	55.76 ± 1.57	93.89 ± 5.00 ^aaaa^	92.38 ± 2.18 ^aaaa^
WC (Z-Score)	−0.52 ± 0.25	5.20 ± 0.61 ^aaaa^	5.36 ± 0.70 ^aaaa^
Weight (kg)	25.17 ± 1.31	54.80 ± 3.88 ^aaaa^	59.58 ± 3.67 ^aaaa^
Weight (Z-Score)	−0.65 ± 0.18	4.28 ± 0.34 ^aaaa^	5.52 ± 0.87 ^aaaa^
Height (cm)	124.25 ± 2.28	138.37 ± 3.15 ^aa^	140.38 ± 2.79 ^aaa^
Height (Z-Score)	−1.07 ± 0.17	1.45 ± 0.31 ^aaaa^	3.11 ± 1.65 ^aaaa^
BMI (kg/m^2^)	16.11± 0.40	27.81 ± 1.20 ^aaaa^	30.16 ± 1.43 ^aaaa^
BMI (Z-Score)	−0.48 ± 0.14	4.12 ± 0.31 ^aaaa^	5.02 ± 0.73 ^aaaa^
Gestational age (weeks)	39.23 ± 0.63	39.34 ± 0.63	39.78 ± 0.55
Newborn weight (g)	3199 ± 152.27	3418 ± 130.88	3155 ± 176.15
Newborn weight (Z-Score)	−0.04 ± 0.22	0.59 ± 0.32	−0.40 ± 0.27 ^b^
Newborn height (cm)	50.14 ± 1.07	51.15 ± 0.72	50.29 ± 1.08
Newborn height (Z-Score)	0.38 ± 0.36	0.80 ± 0.40	−0.18 ± 0.54
Systolic BP (mmHg)	103.90 ± 3.60	118.09 ± 4.584 ^a^	123.50 ± 4.73 ^aa^
Systolic BP (Z-Score)	0.61 ± 0.32	1.49 ± 0.41	1.97 ± 0.41 ^a^
Diastolic BP (mmHg)	63.10 ± 4.76	75.27 ± 2.65 ^a^	77.88 ± 3.38 ^a^
Diastolic BP (Z-Score)	0.43 ± 0.41	1.28 ± 0.22 ^a^	1.38 ± 0.31 ^a^
Biochemical parameters			
Blood glucose curve (mg/dL)			
0 min	85.13 ± 1.41	84.57 ± 1.15	90.70 ± 2.87 ^b^
30 min	n.d.	118.67 ± 11.46	126.40 ± 15.38
60 min	n.d.	114.55 ± 6.08	125.38 ± 11.45
90 min	n.d.	110.45 ± 2.89	122.50 ± 7.47
120 min	n.d.	108.55 ± 3.84	120.75 ± 8.16
Blood insulin curve (µUI/mL)			
0 min	4.84 ± 0.55	11.61 ± 1.05 ^aaaa^	25.29 ± 5.33 ^aa,b^
30 min	n.d.	58.65 ± 10.52	208.84 ± 55.78 ^b^
60 min	n.d.	79.98 ± 13.32	200.65 ± 31.98 ^bb^
90 min	n.d.	60.80 ± 9.15	185.26 ± 23.99 ^bbbb^
120 min	n.d.	47.60 ± 6.95	179.79 ± 31.33 ^bbb^
HOMA-IR	1.03 ± 0.13	2.35 ± 0.21 ^aaaa^	6.81 ± 1.36 ^aaa,bbb^
Total Cholesterol (mg/dL)	155.67 ± 11.06	163.85 ± 9.41	154.40 ± 9.26
Triglycerides (mg/dL)	57.60 ± 7.40	92.15 ± 13.80 ^a^	112.50 ± 19.04 ^aa^
HDL-C (mg/dL)	64.93 ± 6.18	45.73± 2.00 ^aa^	41.00 ± 2.96 ^aaa^
LDL-C (mg/dL)	93.00 ± 6.29	98.91 ± 8.67	91.00 ± 6.83
Creatinine (mg/dL)	0.5 ± 0.02	0.54 ± 0.02	0.59 ± 0.03 ^a^
GOT (U/L)	28.92 ± 1.12	23.09 ± 1.38 ^aa^	24.38 ± 2.12
GPT (U/L)	16.92 ± 1.28	21.67 ± 1.24 ^a^	27.90 ± 5.29 ^a^
Ferritin (mg/mL)	29.82 ± 3.01	37.72 ± 6.11	59.34 ± 8.24 ^aa,b^

Values are shown as the mean ± SEM. ^a^ indicates difference with respect to control (^a^ *p* < 0.05, ^aa^ *p* < 0.01, ^aaa^ *p* < 0.001 and ^aaaa^ *p* < 0.0001) at baseline and ^b^ refers to differences relative to obese children without insulin resistance (ObIR−) (^b^ *p* < 0.05, ^bb^ *p* < 0.01 and ^bbb^ *p* < 0.001, ^bbbb^ *p* < 0.0001). WC, waist circumference; BMI, body mass index; BP, blood pressure; HOMA-IR, homeostasis model assessment of insulin resistance; HDL-C, high density lipoprotein cholesterol; LDL-C, low density lipoprotein cholesterol; GOT, aspartate transaminase; GPT, alanine transaminase.

## Data Availability

Data are available upon request. Data shared are in accordance with consent provided by participants on the use of confidential data.

## Supplementary Materials

The following are available online at https://www.mdpi.com/2076-3921/10/2/244/s1, Figure S1: Glucose and glutathione metabolism in the erythrocyte.
