# Supplementary material for: Blunted Reducing Power Generation in Erythrocytes Contributes to Oxidative Stress in Prepubertal Obese Children with Insulin Resistance
Source: Antioxidants (Basel). 2021 Feb 5;10(2):244. doi: 10.3390/antiox10020244 (PMC7914909; doi:10.3390/antiox10020244)
Supplement: Supplementary file 1 [file antioxidants-10-00244-s001.pdf]

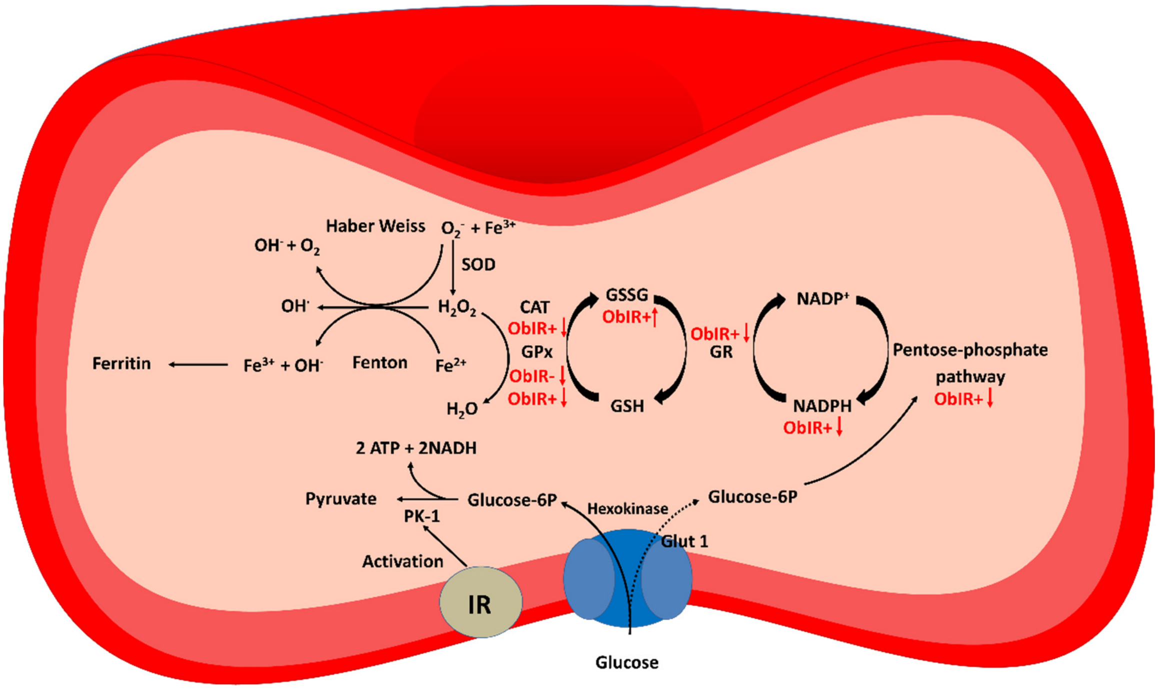

Supplementary figure 1. Glucose and glutathione metabolism in the erythrocyte. Glucose enters the erythrocyte via the insulin-non-dependent glucose transporter Glut 1. Once in the erythrocyte, most of the glucose is derived to the anaerobic glycolysis pathway to produce energy in form of ATP. Alternatively, a small percentage of glucose is metabolized via the pentose-phosphate pathway (PPP) to generate reducing power in form of NADPH after the decomposition of glucose into glucose-6-phosphate and 6-phospho-gluconate catalyzed by glucose-6-phosphate dehydrogenase and 6-phosphogluconate dehydrogenase, respectively. This reducing power obtained via PPP is essential for the correct functioning of the endogenous antioxidant defense, since many enzymes and reduced glutathione availability are NADPH-dependent. An imbalance in reactive oxygen species production and neutralization may lead to oxidative stress, with subsequent biomolecules oxidation. That is why the erythrocyte presents many antioxidant barriers. The anion superoxide is neutralized by Cu-Zn superoxide dismutase (SOD1), producing hydrogen peroxide, that needs to be neutralized by catalase and peroxidases to produce water. Free Fe^{3+} generated by Fenton reaction could be bound to ferritin. Marked in red we indicate the variations found in every marker of this pathway studied in this work. Abbreviations: Cat, catalase; NADPH, nicotinamide adenine dinucleotide phosphate; GPx, glutathione peroxidase; GR, glutathione reductase; GSH, reduced glutathione; GSSG oxidized glutathione; IR, insulin receptor; P, phosphate; SOD, superoxide dismutase. Adapted from Kuhn et al. 2017 [17].
